# New Triterpene Glucosides from the Roots of *Rosa laevigata* Michx

**DOI:** 10.3390/molecules13092229

**Published:** 2008-09-22

**Authors:** Jing-Quan Yuan, Xin-Zhou Yang, Jian-Hua Miao, Chun-Ping Tang, Chang-Qiang Ke, Ji-Bao Zhang, Xiao-Jun Ma, Yang Ye

**Affiliations:** 1 Guangxi Institute of Medicinal Plant, Nanning 530023, P.R. China; E-mails: yjqgx@163.com (J-Q. Y); mjh1962@vip.163.com (J-H. M.); 2 State Key Laboratory of Drug Research, Shanghai Institute of Materia Medica, Chinese Academy of Sciences, Shanghai 201203, P.R. China; E-mails: xinzhou.yang@unibas.ch (X-Z. Y.); tangcp_sh@163.com (C-P. T.); cqke@mail.shcnc.ac.cn (C-Q. K.); 3 Institute of Medicinal Plant Development, Chinese Academy of Medical Sciences and Peking Union Medical College, Beijing 100094, P.R. China

**Keywords:** Ursane-type triterpene glucosides, *Rosalaevigata*, Antifungal activity

## Abstract

Two new ursane-type triterpene glucosides, 2*α*,3*α*,24-trihydroxyurs-12,18-dien-28-oic acid *β*-d-glucopyranosyl ester (**1**) and 2*α*,3*α*,23-trihydroxyurs-12,19(29)-dien-28-oic acid *β*-d-glucopyranosyl ester (**2**), were isolated from the roots of *Rosa laevigata*, together with three known compounds: 2*α*,3*β*,19*α*-trihydroxyurs-12-en-28-oic acid *β*-d-glucopyranosyl ester (**3**), 2*α*,3*α*,19*α*-trihydroxyurs-12-en-28-oic acid *β*-d-glucopyranosyl ester (**4**) and 2*α*,3*β*,19*α*,23-tetrahydroxyurs-12-en-28-oic acid *β*-d-glucopyranosyl ester (**5**). The structures of new compounds were established on the basis of detailed 1D and 2D NMR spectroscopic analyses. Compounds **2** and **5** exhibited modest *in vitro* antifungal activities against *Candida albicans* and *C. krusei*.

## Introduction

*Rosa* *laevigata* Michx. (Rosaceae), an evergreen climbing shrub, is widely distributed throughout southern China [1]. Its fruits, known as a commonly used traditional Chinese medicine (TCM) ‘Jin-Ying-Zi’, are prescribed in the Chinese Pharmacopoeia for the treatment of wet dreams, urinary incontinence, urinary frequency, uterine prolapse, menstrual irregularities and leucorrhea [1,2]. The roots of this plant are used in folk practices of Hunan, Guangdong and Guangxi provinces to cure pelvic inflammation, ascending infection, irregular vaginal bleeding, cervical erosion, and cervicitis [1,3]. In addition, the roots of *R. laevigata* are an essential constituent of three famous proprietary TCMs, i.e., San-Jin-Pian, Jin-Ji-Jiao-Nang, and Fu-ke-Qian-Jin-Pian. These proprietary TCMs focus on the treatment of gynecological infection and diseases of urinary system. No chemical constituent except tannins has been reported from the roots [4,5]. As a part of our *in vitro* antimicrobial screening efforts, the EtOAc fraction of the EtOH extract from the roots of *R. laevigata* showed good antifungal activities against *Candida albicans*, *C. krusei*, and *C. parapsilosis*. Bioassay-guided fractionation led to the isolation of two new ursane-type triterpenoids, 2*α*,3*α*,24-trihydroxyurs-12,18-dien-28-oic acid *β*-d-glucopyranosyl ester (**1**) and 2*α*,3*α*,23-trihydroxyurs-12,19(29)-dien-28-oic acid *β*-d-glucopyranosyl ester (**2**), together with three known compounds, 2*α*,3*β*,19*α*-trihydroxyurs-12-en-28-oic acid *β*-d-glucopyranosyl ester (**3**) [6], 2*α*,3*α*,19*α*-trihydroxyurs-12-en-28-oic acid *β*-d-glucopyranosyl ester (**4**) [7,8] and 2*α*,3*β*,19*α*,23-tetrahydroxyurs-12-en-28-oic acid *β*-d-glucopyranosyl ester (**5**) [7]. Compounds **2** and **5** exhibit modest antifungal activities against *C. albicans* and *C. krusei*. Here, we describe the isolation and structural elucidation of these two new triterpene glucosides, as well as results of antimicrobial tests for all the isolated compounds.

## Results and Discussion

Compound **1** was isolated as an amorphous powder. The molecular formula C_36_H_56_O_10_ was established from the quasi-molecular ion [M+Na]^+^ at m/z 671.3779 in the HR-ESI-MS. The IR absorptions at 3428, 1731 and 1645 cm^-1^ indicated the presence of hydroxyl, carbonyl and olefinic groups, respectively. The UV spectrum showed the absorption of a heteroannular diene at 220 nm [9].

**Figure 1:**
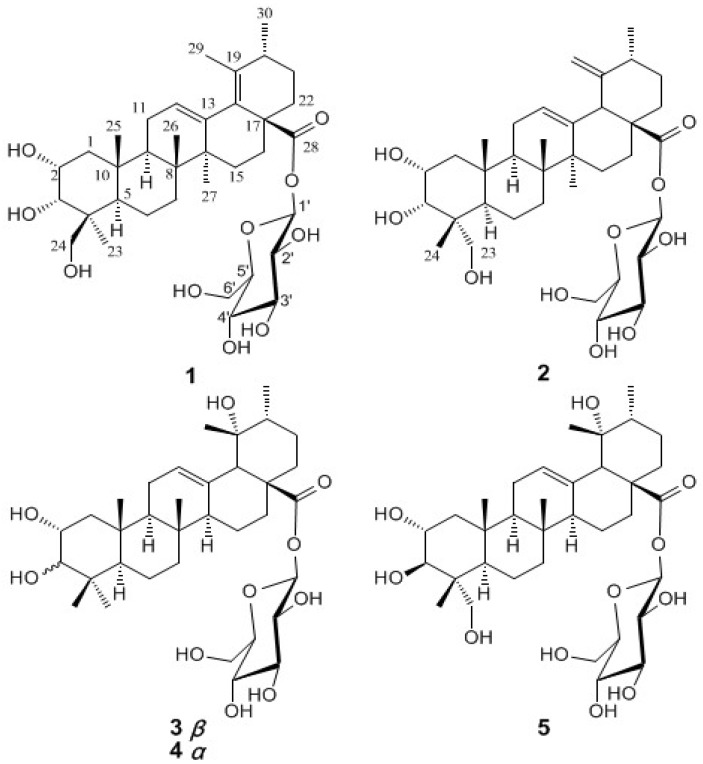
The structures of compounds **1**-**5**.

**Figure 2:**
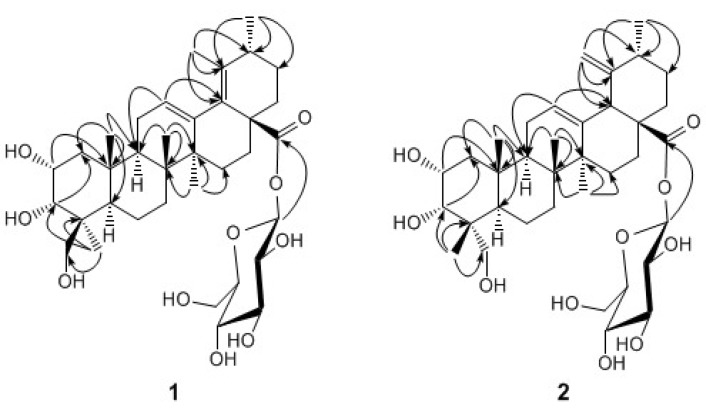
Key HMBC (H→C) correlations for **1** and **2**.

The ^1^H-NMR spectrum of **1** (Table 1) displayed signals corresponding to five tertiary methyls at *δ*_H_ 0.98, 1.05, 1.14, 1.68, and 1.71, a secondary methyl at *δ*_H_ 1.03, an olefinic proton at *δ*_H_ 5.61 (br. s) and oxygenated methine and methylene protons, ascribed to a sugar moiety. The ^13^C-NMR spectrum showed 36 signals, including 6 primary, 10 secondary, 11 tertiary, and 9 quaternary carbons. These NMR data suggested that **1** was a triterpene monoglycoside. A careful analysis of the ^1^H- and ^13^C-NMR data, assigned to the aglycon moiety from its ^1^H-^1^H correlated spectroscopy (^1^H,^1^H COSY), heteronuclear single quantum coherence (HSQC), and ^1^H-detected heteronuclear multiple-bond correlation (HMBC) spectra, suggested that the aglycon was an ursane-type triterpenoid with a heteroannular diene, three hydroxyls and a carboxyl group (C-28). The heteroannular diene was assigned at C-12(13) and C-18(19) by the HMBC correlation from the olefinic proton at *δ*_H_ 5.61 to the carbon at *δ*_C_ 135.2 (C-18), as well as the correlations from the methyls at *δ*_H_ 19.5 and 18.6, assigned to CH_3_-29 and CH_3_-30, respectively, to the same carbon at *δ*_C_ 133.7 (C-19) (Figure 1). Two oxymethine protons at *δ*_H _ 4.46 and 4.60 were observed to correlate to carbons C-10 and C-1, respectively, suggesting that the two hydroxyls were attached to C-2 and C-3. Similarly, the third hydroxyl was determined to be located at C-24 by the HMBC correlation from CH_3_-23 to the oxymethylene carbon (C-24). The sugar moiety was determined to be a D-glucose based on the coupling constants of each proton and the carbon chemical shifts. It was verified by a complete acid hydrolysis with HCl and then comparison with an authentic sample by GC analysis. The chemical shift of the anomeric proton at *δ*_H_ 6.27 (d, *J* = 7.8 Hz) revealed that the glucose was attached to the carbonyl carbon at 174.8 (C-28). This was confirmed by a long-range correlation between the anomeric proton and the carbonyl carbon. The relative stereochemistry of **1** was established by analysis of its coupling constants and ROESY data (Figure 2). The ROESY correlation between CH_3_-23 and H-5 showed the methyl at C-23 was *α*-oriented, and thus the hydroxylmethylene group was in the *β*-orientation. The signal of H-2 was observed as a *ddd* splitting with the coupling constants of 10.5, 4.3 and 3.2 Hz, respectively, indicating a diaxial and two axial-equatorial couplings. Furthermore, the coupling constant of 3.2 Hz between H-2 and H-3 revealed an axial-equatorial coupling. Thus, the orientations of both 2-OH and 3-OH were defined as 2*α*,3*α*, which was confirmed by the ROESY correlations from H-2 and H-3 to CH_2_-24. Therefore, the structure of **1** was determined to be 2*α*,3*α*,24-trihydroxyurs-12,18-dien-28-oic acid *β*-d-glucopyranosyl ester.

**Table 1:** ^1^H- (600 MHz) and ^13^C- (150 MHz) NMR data for **1** and **2** (in Pyridine-*d*_5_).

	1			2	
	*δ*_H_ (*J *Hz)	*δ*_C _		*δ*_H_ (*J *Hz)	*δ*_C_
1	1.84, m; 2.04, m	43.6		1.83, m; 1.93, m	42.7
2	4.46, ddd (10.5, 4.3, 3.2)	66.2		4.27, ddd (9.7, 4.1, 2.7)	66.2
3	4.60, d (3.2)	74.1		4.14, d (2.7)	78.9
4		44.8			42.9
5	1.78, m	48.3		2.03, m	43.6
6	1.43, m; 1.65, m	18.7		1.55, m	18.3
7	1.49, m	35.2		1.38, m	33.1
8		39.6			39.9
9	1.85, m	49.5		2.05, m	49.5
10		38.4			38.4
11	2.04, m	23.9		2.05, m	23.9
12	5.61, br. s	126.5		5.49, br. s	128.4
13		138.6			137.6
14		45.1			41.9
15	1.23, m; 2.41, m	28.9		1.10, m; 2.38, m	29.0
16	1.62, m, 2.57, m	35.5		1.75, m; 1.86, m	25.7
17		50.3			49.8
18		135.2		3.76, s	52.2
19		133.7			153.3
20	2.03, m	34.5		1.83, m	37.5
21	1.23, m; 2.04, m	26.7		1.22, m; 1.37,m	30.7
22	1.67, m; 2.17, m	30.9		1.79, m; 1.94, m	37.1
23	0.98, s	65.1		3.73, d (10.2); 3.88,d (10.2)	71.2
24	3.80, m; 4.12, m	21.9		0.85, s	17.7
25	1.05, s	17.8		1.02, s	17.2
26	1.14, s	18.4		1.14, s	17.4
27	1.68, s	23.8		1.12, s	26.2
28		174.8			176.1
29	1.71, s	19.5		4.95, br. s; 5.10, br. s	110.4
30	1.03, d (7.0)	18.6		1.02, d (7.0)	19.4
Glc					
1'	6.27, d (7.8)	95.9		6.29, d (8.3)	95.9
2'	4.18, dd (8.3, 7.8)	74.0		4.21, dd (8.8, 8.3)	74.0
3'	4.27, m	78.8		4.28, m	78.9
4'	4.35, m	71.1		4.34, dd (9.3, 9.2)	71.1
5'	3.98, m	79.1		4.03, m	79.3
6'	4.37, m; 4.46, m	62.2		4.37, m; 4.47, m	62.2

Compound **2** was also isolated as an amorphous powder. The molecular formula was established as C_36_H_56_O_10_, the same as that of **1**, by the HR-ESI-MS spectrum. Its UV, IR and ^1^H-NMR spectra strongly resembled those of **1**, suggesting that 2 shared the same structural skeleton with **1**. The ^1^H- NMR spectrum of **2** (Table 1) showed the characteristic signals for an exo-methylene at *δ*_H_ 4.95 (br. s) and 5.10 (br. s), instead of a tertiary methyl in **1**, which indicated that the double bond was transferred from C-18(19) to C-19(29) [10]. Another difference observed was the chemical shift value of C-23, which downfield shifted to *δ*_C_ 71.2 in **2** instead of *δ*_C_ 65.1 in **1**. This evidence suggested that the configuration of CH_3_-24 in **2** might be opposite to that in **1**. The relative stereochemistry of **2** was also established by analysis of its coupling constants and ROESY data (Figure 2). The ROESY correlations (Figure 2) between CH_2_-23 at *δ*_H_ 3.88 (d, *J* = 10.2 Hz) and 3.73 (d, *J* = 10.2 Hz) and H-5 at *δ*_H_ 2.03 (m) revealed that the hydroxylmethylene exhibited *α*-oriented, and CH_3_-24 was then *β*-oriented. Signals corresponding to H_2_-1, H-2 and H-3 showed the similar chemical shifts and the same multiplicities as **1** in the ^1^H-NMR spectrum, indicating that **2** has the same 2*α*,3*α* oriented hydroxyls as **1**. The ROESY correlations from H-2 and H-3 to CH_3_-24 further supported this stereochemistry assignment. Complete acid hydrolysis with HCl yielded d-glucose, which was determined by GC analysis. Thus, the structure of **2** was established as 2α,3α,23-trihydroxyurs-12,19(29)-dien-28-oic acid *β*-d-glucopyranosyl ester.

The known compounds **3**-**5** were identified by comparison with the NMR and MS data with the literature values [6,7,8].

### Biological activity

All the isolates were subjected to the dilution assay for *in vitro* antimicrobial activities against *Staphylococcus aureus* (ATCC 25923), *S. epidermidis* (ATCC 26069), *Bacillus subtilis* (ATCC 6633), *Escherichia coli* (ATCC 25922), *Candida albicans* (ATCC 64550), *C. krusei* (ATCC 6258), *C. parapsilosis* (ATCC 22019), *Klebsiella pneumoniae*, *Torulopsis glabrata*, and *Cryptococcus neoformans*. The tests were carried out according to the protocols described in the literature [11]. *K. pneumoniae*, *T. glabrata* and *C. neoformanin* were obtained from Huashan Hospital, Shanghai, P. R. China. Two antimicrobial agents, chloroamphenicol and fluconazole, were used as positive controls in these tests. Among the tested compounds, compounds **2** and **5** showed modest antifungal activities against *C. albicans* and *C. krusei* with MIC 12.5–25 μg/mL (Table 2). It was observed that the presence of the hydroxymethylene group at C-23 in the ursane-type triterpenoid has a substantial contribution to the antifungal activity. Compounds **2** and **5** (containing such a 23α-hydroxymethylene group) show stronger antifungal activity than compound **1** (24*β*-hydroxymethylene group) or compounds **3** and **4** (without such functional groups at C-23 positions).

**Table 2:** MIC^a^ Values of **1**-**5** for Antimicrobial Activities (μg/mL).

	1	2	3	4	5	Chloroamphenicol	Fluconazole
*S. aureus*	100	100	>200	>200	>200	4.0	
*S. epidermidis*	>200	>200	100	>200	>200	4.0	
*B. subfitis*	100	50	100	>200	>200	8.0	
*E. coli*	>200	100	>200	>200	>200	2.0	
*K. pneumoniae*	>200	100	>200	>200	>200	1.5	
*C*.*albicans*	100	12.5	100	100	25		1.56
*C. krusei*	50	12.5	50	>200	12.5		50
*C. parapsilosis*	>200	50	100	>200	200		1.56
*T. glabrata*	>200	>200	>200	>200	>200		6.25-12.5
*C. neoformans*	>200	>200	>200	>200	>200		50

^a^ MIC was defined as the lowest concentration that inhibited visible growth.

Pentacyclic triterpenoids are distributed widely in plants and reported to exhibit extensive bioactivities, such as antimicrobial, anti-tumor, and anti-HIV properties. In this study, ursane-type triterpene glucosides **1**-**5** were identified from the roots of *R. laevigata*, and compounds **2** and **5** showed moderate antifugal activities. As the main components, they can account for the bioactivity of the EtOAc extract to some extent. These compounds are the chemical constituents reported for the first time from this part of *R. laevigata* except for tannins. They can be further considered as the chemical fingerprints of this folk medicine.

**Figure 3:**
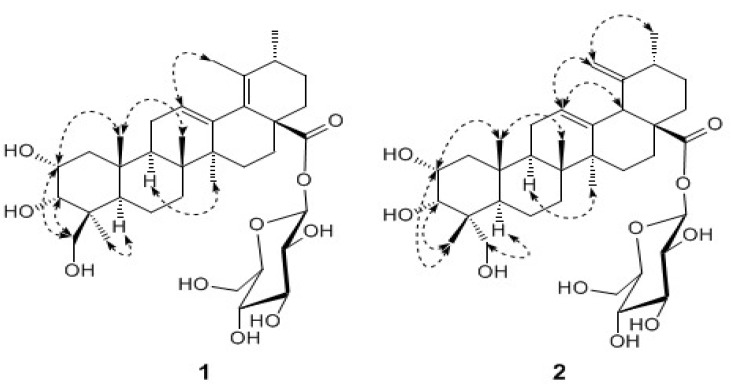
Key ROESY (↔) correlations for **1** and **2**.

## Experimental

### General

Column chromatography (CC): silica gel (Qing Dao Hai Yang Chemical Group Co.; 200-300 and 300-400 mesh), polyamide resin (Tai Zhou Si Jia Sheng Hua Chemical Group Co., 100-200 mesh), and MCI-gel CHP20P (75-150 *μ*m, Mitsubish Chemical Industries, Ltd.). TLC and preparative TLC: precoated silica gel plates (Yan Tai Zi Fu Chemical Group Co.; GF-254). Preparative and Semi-preparative HPLC system: two PrepStar SD-1 solvent delivery modules, a ProStar UV-Vis 320 detector and a ProStar 701 Fraction Collector (Varian, Walnut Creek, CA, USA); a LiChrospher 100 RP-18 (Merck, Darmstadt, Germany) column (220 × 25 mm i.d., 12 μm) was used for preparative isolation. M.p.: Fisher-Johns melting point apparatus; uncorrected. Optical rotation: Perkin-Elmer 341 polarimeter. UV Spectra: Hewlett-Packard 8452A diode array spectrophotometer, λ_max_ in nm. IR Spectra: Nicolet Magna-FT-IR-750 spectrometer, *ν*_max_ in cm^-1^. ^1^H- and ^13^C-NMR Spectra: Bruker DRX-400 and Varian Unity Inova 600 MHz spectrometers; chemical shifts *δ* in ppm, with residual Pyridine-*d*_5_ as internal standard, coupling constant *J* in Hz, assignments supported by ^1^H,^1^H COSY, HSQC, ROESY and HMBC experiments. ESI-MS and HR-ESI-MS: Q-TOF Micro mass spectrometer in *m/z*. Gas chromatography: Shimadzu GC 14-BPF apparatus equipped with a 5% OV225/AW-DMCS-Chromosorb W (80—100 mesh) column (2.5m × 3mm) and a hydrogen-flame ionization detector.

### Plant Material

The dried roots of *R. laevigata* were purchased from Nanning Yixin Pharmaceuticals Ltd., Guangxi, P.R. China in September 2006, and identified by Prof. Chao-liang Zhang of Guangxi Botanical Garden of Medicinal Plants. A voucher specimen (2006047) was deposited in the Herbarium of Shanghai Institute of Materia Medica, Chinese Academy of Sciences.

### Extraction and Isolation

Dried roots of *R. laevigata* (3.0 kg) were mechanically powdered and percolated with 95% EtOH three times (5 L each) at room temperature. The extract was filtered and concentrated *in* *vacuo* (40°C) to give an EtOH extract (440 g). The extract was suspended in water, and then partitioned successively with petroleum ether (Pe, b.p. 60-90°C), CHCl_3_, EtOAc, and *n*-BuOH to afford the Pe (15.6 g), CHCl_3_ (22.0 g), EtOAc (223.5 g) and *n*-BuOH fractions (120.0 g), respectively. The EtOAc fraction (30 g) was subjected to CC over polyamide resin (500 g) and eluted with 40%, 60%, 80% and 100% aqueous EtOH in a step manner. The 40% EtOH fraction was subjected to CC over MCI gel (100 mL) and eluted with 50%, 60%, 70% and 80% aqueous EtOH to afford subfractions 1.1-1.4. The subfraction 1.2 was chromatographied on a silica gel column and eluted with CHCl_3_/MeOH (7:1) to yield **3** (300 mg) and **4** (124 mg). The subfraction 1.3 afford **5** (85 mg) by CC over silica gel eluted with CHCl_3_ /MeOH (10: 1). The 60% EtOH fraction was subjected to a Sephadex LH-20 column and eluted with MeOH/CHCl_3_ (3: 1) to give subfractions 2.1-2.5. The subfraction 2.2 was purified by prep. TLC with CHCl_3_ /MeOH (8: 1) to yield **1** (15 mg). The subfraction 2.4 was submitted to preparative HPLC (CH_3_CN in H_2_O from 15% to 70%, 150 min) to yield **2** (11 mg, *t_R_* 95 min). 

*Compound **1***: Amorphous powder, 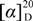 + 82.1 (*c* = 0.5, MeOH); IR (KBr) cm^-1^: 3428, 2935, 1731, 1645, 1457, 1073, 1030; HR-ESI-MS *m/z*: 671.3779 [M+Na]^+^ (Calcd for C_36_H_56_ NaO_10_, 671.3771), ^1^H- and ^13^C-NMR: see Table 1.

*Compound **2***: Amorphous powder, 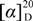 + 72.4 (*c* = 0.5, MeOH); UV *λ*_max_ (MeOH) nm: 220; IR (KBr) cm^-1^: 3417, 2945, 1716, 1632, 1442, 1064, 1029; HR-ESI-MS *m/z*: 671.3768 [M+Na]^+^ (Calcd for C_36_H_56_ NaO_10_, 671.3771); ^1^H- and ^13^C-NMR: see Table 1. 

### Determination of the Sugar Components [10]

Compounds **1**–**2** (4 mg) in 10% HCl soln./dioxane (1:1, 1 mL) was heated separately at 80 °C for 4 h in a water bath. The mixture was neutralized with Ag_2_CO_3_, filtered, and then extracted with CHCl_3_ (30 mL). The aqueous layer was evaporated, and then the residue was treated with L-cysteine methyl ester hydrochloride (4 mg) in pyridine (0.5 mL) at 60 °C for 1 h. After reaction, the solution was treated with acetic anhydride (3 mL) at 60 °C for 1 h. Authentic samples were prepared by the same procedure. The acetate derivatives were subjected to GC analysis to identify the sugars (column temperature 210 °C; injection temperature 250 °C; carrier gas N_2_ at a flow rate of 25 mL/min). D-glucose (*t_R_* 1.8 min) was observed from **1** and **2**.

### Antimicrobial activity

Ths was determined by the broth dilution technique as previously described [11]. The solutions (maximum concentration) of the compounds (i.e. the compounds that induced zones of inhibition) were prepared in DMSO, serially (2-fold) diluted and 0.5 mL of each dilution was introduced into a test tube containing 4.4 mL of Selenite broth; then 0.1 mL of microbial suspension (5 × 10^5^ cfu/mL) was added and the mixture was homogenized. The total volume of the mixture was 5 mL, with the test compound concentrations in the tube ranging from 200 to 12.5 μg/mL and those of the standard compounds, i.e. Chloroamphenicol and Fluconazole, ranging from 8.0 to 2.0, and 50 to 1.56 μg/mL, respectively. After 24 h of incubation at 37 °C, the MIC was reported as the lowest concentration of a compound that prevented visible growth.
